# Abnormal Brain Functional Connectivity of the Hypothalamus in Cluster Headaches

**DOI:** 10.1371/journal.pone.0057896

**Published:** 2013-02-27

**Authors:** Enchao Qiu, Yan Wang, Lin Ma, Lixia Tian, Ruozhuo Liu, Zhao Dong, Xian Xu, Zhitong Zou, Shengyuan Yu

**Affiliations:** 1 Department of Neurology, Chinese PLA General Hospital, Beijing, People's Republic of China; 2 Department of Radiology, Chinese PLA General Hospital, Beijing, People's Republic of China; 3 Department of Neurology, The First Affiliated Hospital of Chinese PLA General Hospital, Beijing, People's Republic of China; 4 Department of Biomedical Engineering, School of Computer and Information Technology, Beijing Jiaotong University, Beijing, People's Republic of China; Beijing Institute of Radiation Medicine, China

## Abstract

The aim of this study was to detect the abnormality of the brain functional connectivity of the hypothalamus during acute spontaneous cluster headache (CH) attacks (*‘in attack’*) and headache-free intervals (*‘out of attack’*) using resting-state functional magnetic resonance imaging (RS-fMRI) technique. The RS-fMRI data from twelve male CH patients during *‘in attack’* and *‘out of attack’* periods and twelve age- and sex-matched normal controls were analyzed by the region-of-interest -based functional connectivity method using SPM5 software. Abnormal brain functional connectivity of the hypothalamus is present in CH, which is located mainly in the pain system during the spontaneous CH attacks. It extends beyond the pain system during CH attack intervals.

## Introduction

Cluster headache (CH) is one of the most distinctive primary headache disorders [1]. It is an extremely painful headache disorder with a prevalence of 0.1–0.4%, occurring predominantly in males, with a 9:1 ratio of males to females [2]. The prominent clinical features of the disorder are extremely severe unilateral pain (usually retro-orbital) and ipsilateral cranial autonomic symptoms such as conjunctival injection and lacrimation [1]. The headaches usually occur in cluster periods that last for several weeks or months and are separated by remission periods that usually last for several months or years [1]. During a cluster period, patients may experience as few attacks as one every other day to as many as eight per day, lasting 15–180 minutes per attack [1]. These characteristics led to the idea that CH is probably a disorder that originates in an abnormal brain function [3].

During the past ten years, functional neuroimaging studies have played an important role in exploring the brain functional changes in CH. Positron emission tomography (PET) studies of CH that was triggered with nitroglycerin (NTG) have suggested that the ipsilateral hypothalamus plays an important role as the CH generator [4], [5]. The hypothalamic involvement in the genesis of CH attacks has been further supported by alteration of the same brain area using voxel-based morphometric MRI [6], by low N-acetylaspartate/creatine and choline/creatine levels in the hypothalamus in patients with episodic CH using 1H-MR spectroscopy [7], and by a beneficial effect of deep brain stimulation of this area [8]–[11]. Other brain regions, such as the anterior cingulate cortex (ACC), the posterior cingulate cortex (PCC), the orbitofrontal cortex and insular cortex related to pain processing have also been considered to be related to the CH using the PET [5] and resting-state functional MRI (RS-fMRI) [12]. Another recent study with diffusion tensor imaging (DTI) has revealed patients who have episodic CH have microstructural brain changes in regions that belong to the pain matrix [13]. Furthermore, Morelli N et al. [14] investigated the cerebral activation center during spontaneous attack in four patients who had CH with fMRI using the Brain Voyager QX version 1.7.81 software package. A significant activation of the hypothalamus ipsilateral to the headache side was indicated. In addition, an RS-fMRI study by Rocca MA et al. has more recently reported that a diffuse abnormality of brain functional connectivity that extends beyond the antinoceptive system is present in CH patients outside the cluster period by use of an independent component analysis (ICA) and a seed correlation analysis [15]. However, to our knowledge, few studies, have ever detected the functional connectivity of the hypothalamus in CH patients during spontaneous CH attacks and CH attack intervals in the cluster period by using RS-fMRI.

RS-fMRI has recently attracted further attention because participants are instructed simply to remain motionless and to keep their eyes closed during the fMRI scan [16], [17]. Resting-state functional connectivity (rs-FC) is a descriptive measure of spatiotemporal correlations between distinct cerebral regions [18], [19]. It can be extracted from spontaneous activity of the resting brain. There is a high temporal coherence of low-frequency (<0.08 Hz) fluctuations (LFFs) in resting-state fMRI time series between spatially functionally related brain regions. These LFFs in MR signal intensity are supposed to arise from fluctuations in capillary blood flow and blood oxygenation, which are secondary to neuronal activity [16], [20]. The presence of underlying neural connections is implied [21]. Rs-FC has been used to investigate the pathophysiology of neuropsychiatric disorders, such as Alzheimer's disease [22], attention deficit hyperactivity disorder [23], and schizophrenia [24]. Together, these studies have suggested that the rs-FC might be useful in revealing the pathophysiology of neuropsychiatric disorders in the resting-state.

In the present study, we used a region-of-interest (ROI)-based method to determine whether the abnormal rs-FC between the hypothalamus and some pain-related regions or some other brain regions are present in CH patients during acute spontaneous CH attacks and CH attack intervals in the cluster period. Detection of abnormal rs-FCs of the hypothalamus in these groups revealed three critical findings. They are that: (1) there is an increased rs-FC between the hypothalamus and brain regions related to pain processing such as the ACC and the PCC during spontaneous CH attacks, (2) there is an altered rs-FC between the hypothalamus and brain regions beyond that is related to emotional modulation of pain during CH attack intervals, and (3) these regions have relatively high sensitivity and specificity.

## Materials and Methods

### Subjects

Twelve right-handed male patients with episodic CH (aged 19–50 years) were included in the study. All suffered from a right-sided headache. Each patient met the criteria for CH, according to the second edition of the international classification of headache disorders (ICHD-Π) [1]. The clinical characteristics are summarized in detail in Table 1. None of the patients had taken prophylactic medication before enrolling in the study. Patients with other neurological or psychiatric and systemic conditions were excluded. Twelve right-handed, healthy, male volunteers (of age 19–51 years) who had no history of primary headache syndrome or other chronic pain conditions served as normal controls. The study protocol was approved by the Ethical Committee of the Chinese PLA General Hospital and complied with the Declaration of Helsinki. Prior to the fMRI scans, all participants were informed of the purpose and methods of this study. A written informed consent was obtained from each participant.

**Table 1:** Clinical characteristics of the 12 episodic cluster headache patients.

Patient	Sex	Age (years)	Affected side	Disease duration (years)	Frequency of attacks (per day)	Typical duration of attacks (min)	Typical duration of cluster period (weeks)	VAS pain rating for attacks (/10)
1	M	41	R	20	1–3	15–120	8–12	10
2	M	19	R	3	0.5–3	30–120	20	9
3	M	23	R	5	1	120	1–2	7
4	M	50	R	30	1–3	120–180	4–8	8
5	M	32	R	16	1–4	120	4–8	9
6*	M	24	R	-	0.5	30–60	-	8.5
7	M	26	R	10	0.5–3	180–240	3–4	7.5
8	M	34	R	16	4	60–120	2–4	10
9	M	29	R	10	1–2	60–120	4–8	7.5
10	M	28	R	8	3–4	30–60	8–12	7.5
11	M	46	R	27	1	120	4	10
12	M	44	R	20	1–3	30–60	4–8	10

* The patient experienced the first cluster period.

M, male; R, right; VAS, visual analogue scale.

### Image Acquisition

Two fMRI scans of each patient were successfully acquired – one during the acute spontaneous attack (*‘in attack’*) and another at least four hours after the previous attack, but before the next attack (*‘out of attack’*). Both scans were acquired during the cluster period. No drugs were provided before the first fMRI scan. Immediately after the first fMRI scan, the CH patients were given 100 percent oxygen, which they inhaled by means of a tight-fitting mask at a flow rate of 8 to 10 liters/min for 15 minutes. All patients gradually achieved headache remission. Each healthy male volunteer was scanned once in a resting state.

RS fMRI scans were required on a GE Signa 3.0T scanner (General Electric, Milwaukee, Wisconsin, USA). Functional images were collected axially using an echo planar imaging (EPI) sequence (echo time [TE] = 30 ms, repetition time [TR] = 2000 ms, flip angle [FA] = 90°, field of view [FOV] = 22 cm, matrix = 64×64, slices = 32, thickness = 3.0 mm). The total fMRI scanning time was 369 seconds. One hundred and eighty phases were acquired for each scan, not including the initial phases. The initial phases were discarded by the scanner to reduce the influence of the instability of the initial MRI signal and to allow the subjects to adapt to the environment. All subjects were instructed to close their eyes, to remain motionless, and to think of as little as possible throughout the resting-state scanning. A conventional MRI was acquired for each subject to exclude the brain structural lesions.

### Image Preprocessing

RS fMRI data were first preprocessed using the statistical parametric mapping (SPM5, http://www.fil.ion.ucl.ac.uk/spm). The 180 images were corrected for the acquisition time delays between different slices and realigned to the first volume to correct for head motion. The realigned images were further spatially normalized to the Montreal Neurological Institute EPI template with a data re-sampling to 3 mm cubic voxels, followed by spatial smoothing with a Gaussian Kernel of 4 mm×4 mm×4 mm full-width at half maximum. Then, to reduce spurious variances that were unlikely to reflect neuronal activity, several additional preprocessing steps were undertaken. These included regression of the constant elements and linear drift of signals, regression of six parameters obtained by head motion correction and regression of the average of the signals from white matter, CSF and 12 region of interest on the exterior of the brain as the intrinsic noise of the scanner. Finally, the fMRI time series were band-pass filtered between 0.01–0.08 Hz to remove low-frequency drifts and physiological high-frequency noises.

### Rs-FC Analysis and Statistics

Correlations between the hypothalamus and other brain regions were examined using a region-of-interest (ROI)-based method, which is most commonly used to detect the functional connectivity of a specific brain region by selecting this region as a seed and evaluating the correlation between the seed region and every other voxel of the brain. A PET study by May A, et al. showed that the Talairach coordinates [–2, –18, −8] that were located at the left hypothalamus had the largest peak z-score in the CH patients who had a left-sided headache [4]. Also, many previous neuroimaging studies have demonstrated that the hypothalamus ipsilateral to the headache side is involved in the pathogenesis of CH attacks [5]–[7], [14]. Thus, in the present study of CH patients who have a right-sided headache, the right hypothalamus was selected as the ROI, and defined as the 6-mm-radius spheres that were centered at Talairach coordinates [2, –18, −8]. The mean time series of the ROI was defined as the seed reference time course for further correlation analysis.

A paired *t*-test was carried out to compare the functional connectivity maps of the CH patients during the ‘*in attack*’ and *‘out of attack’* periods. A random effect, two-sample *t*-test was performed to compare the CH patients during the ‘*out of attack*’ periods and normal controls. These statistical results were used to determine the brain regions that show significant differences in correlation to the right hypothalamus. The resulting statistical maps were corrected by Monte Carlo simulations using the AFNI AlphaSim program (parameters were: individual voxel P value = 0.05, voxels = 54. Also, see http://afni.nimh.nih.gov/pub/dist/doc/manual/AlphaSim.pdf) [25]. This yielded a corrected threshold of P<0.05 with a minimum volume of 1458 mm^3^.

To provide a clear idea of the functional connectivity differences between the groups, we plotted a scattergram for each region that exhibited significant between-group differences. Specifically, brain regions that exhibited significant between-group differences were selected as ROIs. In case of any confusion, these ROIs are designated below as FC-ROIs. The mean functional connectivity with the right hypothalamus of all voxels within each FC-ROI was then calculated for each individual. For each FC-ROI, the mean functional connectivity with the right hypothalamus of individual subject was then plotted as a scattergram.

## Results

### Rs-FC differences between ‘in attack’ and ‘out of attack’

**Figure 1:**
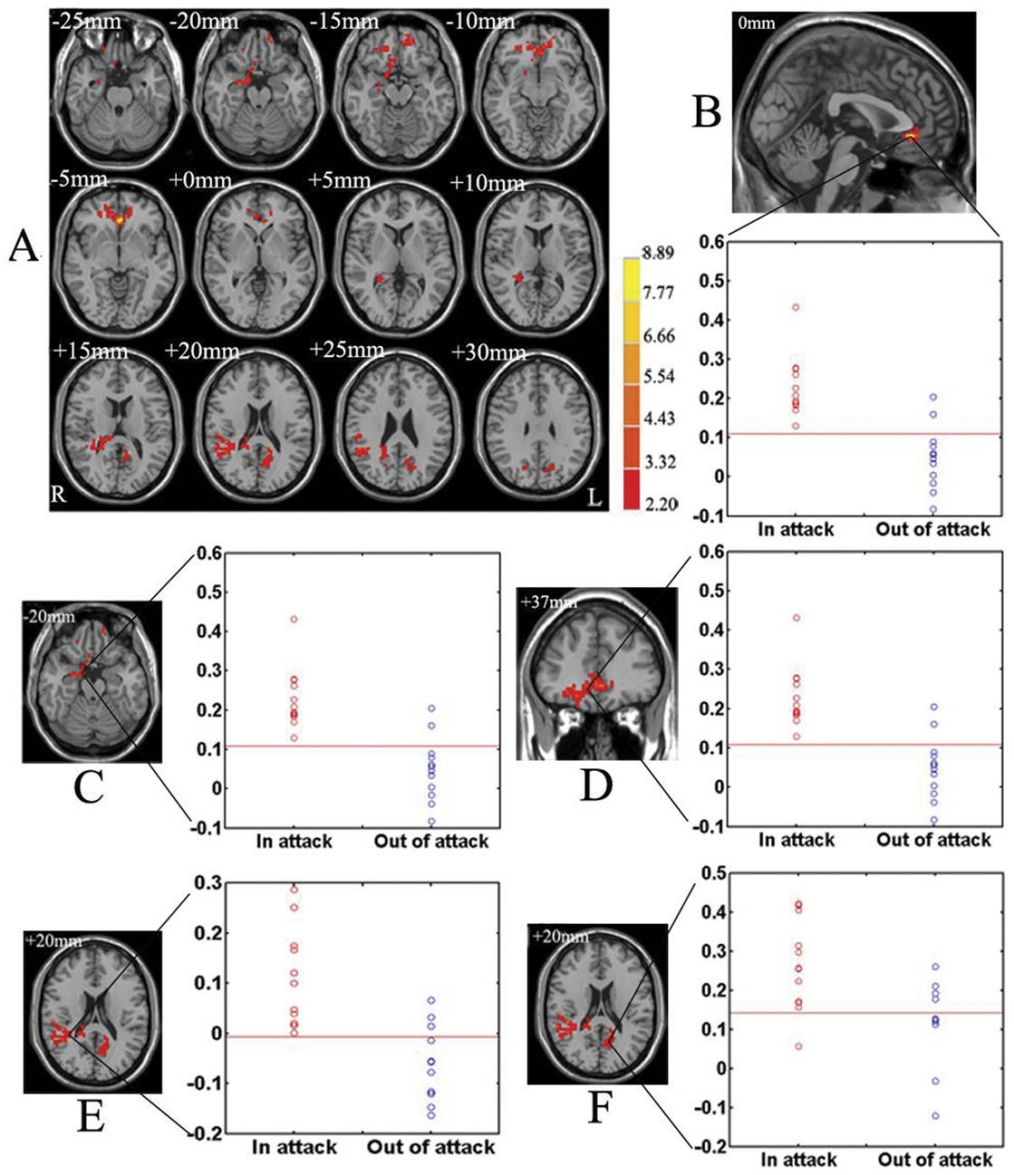
Altered functional connectivity of the hypothalamus and the mean functional connectivity with the right hypothalamus for each subject in CH patients during the *‘in attack’* periods vs. those during the *‘out of attack’* periods. (A) Brain regions showing significantly increased functional connectivity with the right hypothalamus in CH patients during the *‘in attack’* periods in comparison to those during the *‘out of attack’* periods, thresholded at P<0.05, corrected by Monte Carlo simulations. Color bar indicates the *t*-score. *R*, right; *L*, Left. Brain regions in three dimensions including the bilateral anterior cingulate cortex (B), the right parahippocampal gyrus, hippocampus and amygdale (C), and the bilateral frontal cortex (D), together with other brain regions such as the right angular, supramarginal gyrus, insula, superior temporal gyrus and precuneus (E), and the left precuneus, parietal lobe, posterior cingulate cortex and occipital lobe (F) showing the corresponding scattergram of the mean functional connectivity with the right hypothalamus for each subject in CH patients during the *‘in attack’* and *‘out of attack’* periods.

In the paired *t*-test statistical analysis, significant increases of functional correlation to the right hypothalamus were detected in CH patients during the *‘in attack’* periods in comparison to those during the *‘out of attack’* periods in three clusters. The clusters included the brain regions such as the ACC, the PCC, the superior frontal gyrus (SFG), the middle frontal gyrus (MFG), the inferior frontal gyrus (IFG), the superior temporal gyrus (STG), the inferior parietal Lobule (IPL), the parahippocampal gyrus and the amygdala (P<0.05, corrected). We selected the three clusters as three FC-ROIs. Three scattergrams of the mean functional connectivity with the right hypothalamus for each subject in the two groups are shown in Fig 1 (B, C, D, and E, and F). Taking the FC-ROI at the cluster including the bilateral ACC, the right parahippocampal gyrus, hippocampus, amygdale, and the bilateral frontal cortex (Fig 1. B, C, D) as an example: the means differed significantly using a two-sample t test (T = 6.1162, and P = 7.5615e-005). Using a cutoff of 0.1094, the test yields a sensitivity of 100% and a specificity of 83.33% in distinguishing CH patients during *‘in attack’* period from those during an *‘out of attack’* period. Further details are shown in Fig 1 and Table 2.

**Table 2:** Increased functional connectivity of the hypothalamus in CH patients during the *‘in attack’* periods vs. those during the *‘out of attack’* periods.

Clusters	Brain regions	Hemis-phere	BA	Numb-er of voxels	Talairach coordinates (mm)			*t* score (peak)	Cutoff point	Specifi-city	Sensiti-vity	T score	*P* value
					x	y	z						
1	ACC/SFG/MFG/IFG/RG/OG//Parahippo-campal gyrus/Hip-pocampus/Amygdala	B//R	32/12/10/11/11/11/34/-/-	336	0	32	-7	8.8856	0.1094	0.8333	1	6.1162	7.5615e-005
2	Angular/Supramar-ginal gyrus/Insu-la/STG/Precuneus	R	39/40/-/22/-	209	15	-36	18	5.2732	-0.0064	0.7500	1	6.0408	8.4193e-005
3	Precuneus/Parietal lobe/PCC/Occipital lobe/	L	-/7/-/31/19	77	-12	-69	28	3.7087	0.1414	0.6667	0.9167	4.1568	0.0016

Threshold: *P*<0.05 (corrected by Monte Carlo simulation).

R, right; L, left; B, bilateral; BA, Broadmann Area; ACC, anterior cingulate cortex; SFG, superior frontal gyrus; MFG, middle frontal gyrus; IFG, inferior frontal gyrus; RG, rectal gyrus; OG, orbital gyrus; STG, superior temporal gyrus; PCC, posterior cingulate cortex; -, no BA.

### Analysis of CH Patients During ‘out of attack’ vs. Normal Controls

**Figure 2:**
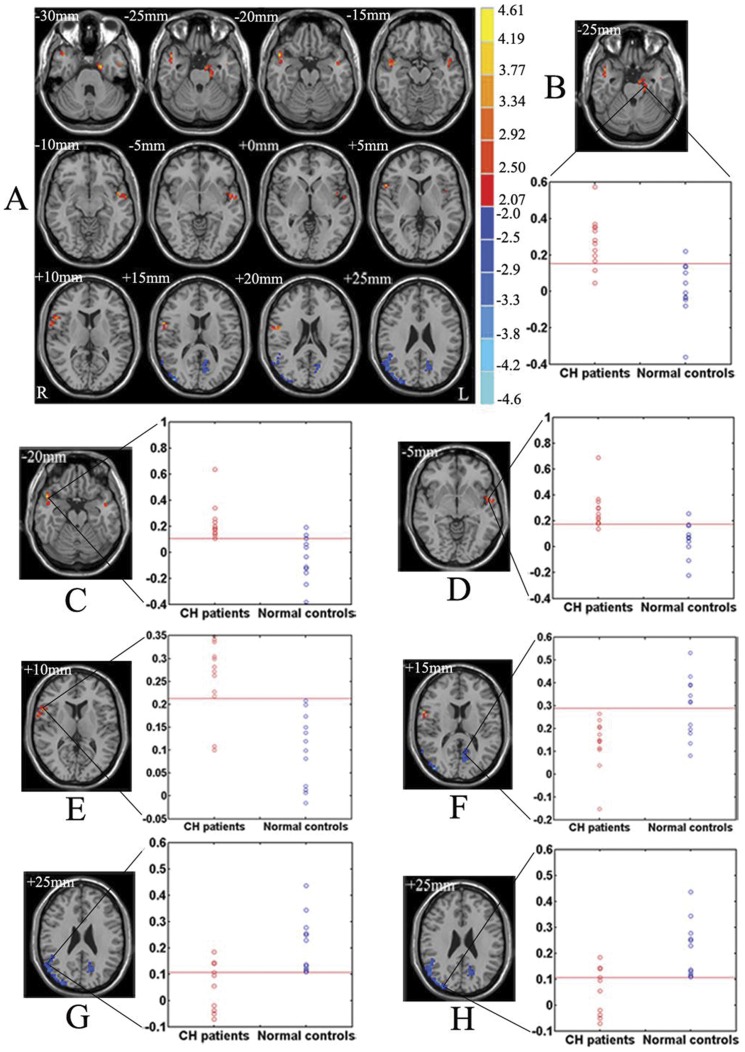
Altered functional connectivity of the hypothalamus and the mean functional connectivity with the right hypothalamus for each subject in CH patients during the *‘out of attack’* periods vs. that in normal controls. (A) Brain regions showing significantly increased (*t*-score>0) or decreased (*t*-score<0) functional connectivity with the right hypothalamus in CH patients during the *‘out of attack’* periods compared to that in normal controls, thresholded at P<0.05, corrected by Monte Carlo simulations. Color bar indicates the *t*-score. *R*, right; *L*, Left. Brain regions including the left parahippocampal gyrus and uncus (B), the right superior temporal gyrus, middle temporal gyrus and temporal pole (C), the left superior temporal gyrus, middle temporal gyrus, temporal pole, insula and fusiform gyrus (D), the right precentral gyrus and inferior frontal gyrus (E), the left occipital lobe, precuneus, inferior parietal lobule and cuneus (F), the right superior temporal gyrus, middle temporal gyrus and superior parietal lobule (G), and the right occipital lobe, precuneus, inferior parietal lobule and cuneus (H) showing the corresponding scattergram of the mean functional connectivity with the right hypothalamus for each subject in CH patients during the *‘out of attack’* periods and normal controls.

In the two-sample, *t*-test, statistical analysis, a significantly increased functional correlation to the right hypothalamus was detected in CH patients during the *‘out of attack’* periods compared to normal controls in four clusters. The clusters included the brain regions such as the IFG, the STG, the middle temporal gyrus (MTG), the temporal pole (TP), the insular cortex, the parahippocampal gyrus and the uncus (P<0.05, corrected). Decreased functional correlation to the right hypothalamus was also detected in three clusters, including brain regions such as the precuneus, the IPL, and the occipital lobe (P<0.05, corrected). We selected the seven clusters as seven FC-ROIs. Seven scattergrams of the mean functional connectivity with the right hypothalamus for each subject in the two groups are shown in Fig 2 (B, C, D, E, F, G, and H). Taking the FC-ROI at the cluster including the right precentral gyrus and IFG (Fig 2. E) as an example, the means were differed significantly using a two-sample t test (T = 7.4779, and *P* = 1.2336e-005). Using a cutoff of 0.2128, the test yields a sensitivity of 83.33% and a specificity of 100% in distinguishing CH patients during an *‘out of attack’* period from normal controls. Further details appear in Fig 2 and Table 3.

**Table 3:** Altered functional connectivity of the hypothalamus in CH patients during the *‘out of attack’* periods vs. that in normal controls.

Clusters	Brain regions	Hemis-phere	BA	Number of voxels	Talairach coordinates (mm)			*t* score (peak)	Cutoff point	Specifi-city	Sensiti-vity	T score	*P* value
					x	y	z						
1	Precentral gyrus/IFG	R	6/11	56	59	6	11	4.1824	0.2128	1	0.8333	7.4779	1.2336e-005
2	STG/MTG/TP	R	22/21/38/-	60	50	11	-18	4.1772	0.1046	0.8333	1	3.8229	0.0028
3	Parahippo-campal gyrus/Unc-us	L	34/35	59	-15	-7	-22	4.0942	0.1545	0.9167	0.8333	4.5910	7.7628e-004
4	STG/MTG/TP/Insula/Fusiform gyrus	L	22/21/38/-/37	62	-48	2	-10	3.7742	0.1759	0.9167	0.9167	3.1257	0.0097
5	STG/MTG/SPL	R	22/21/7	128	56	-59	42	-4.6685	0.1073	1	0.6667	-4.0593	0.0019
6	Occipital lobe/Precuneus/IPL/Cuneus	R	19/-/40/17	89	42	-84	18	-3.9707	0.1203	0.7500	0.9167	-3.2896	0.0072
7	Occipital lobe/Precuneus/IPL/Cuneus	L	19/-/40/17	67	-18	-66	28	-3.6195	0.2895	0.5833	1	-2.9203	0.0139

Threshold: *P*<0.05 (corrected by Monte Carlo simulation).

*t*-score >0 and *t*-score <0 respectively signify the brain region had increased and decreased functional connectivity with the right hypothalamus in CH patients during the ‘*out of attack*’ period vs. normal controls.

R, right; L, left; BA, Broadmann Area; IFG, inferior frontal gyrus; STG, superior temporal gyrus; MTG, middle temporal gyrus; TP, temporal pole; SPL, superior parietal lobule; IPL, inferior parietal lobule.

## Discussion

Due to advanced methods like PET and fMRI, remarkable progress toward unraveling the mystery of CH has been seen over the past decade. Functional imaging with PET has confirmed a highly specific activation of the hypothalamic grey matter in NTG-triggered CH attacks [4], [5]. Based on RS-fMRI, Morelli N et al. reported significant hypothalamic *activation* of the hypothalamus ipsilateral to the pain side in CH patients *during acute spontaneous attacks* [14], and Rocca MA et al. reported *resting-state network abnormalities* in CH patients *outside the cluster period* [15]. Using *the ROI-based rs-FC* method, the present study, which is based on RS-fMRI, has revealed the altered functional correlations of hypothalamus in CH patients *during different states in the cluster period*. The findings provide a better understanding of the neuroanatomical and pathophysiological basis of the conditions.

In this study, we analyzed the RS temporal coherence of LFFs between hypothalamus and other brain regions in CH patients to gain additional insight into the abnormal brain functional connectivity that are associated with this condition. To understand the changes during spontaneous CH attacks, we studied patients in the acute spontaneous headache attack state in comparison to those in the headache attack remission state. Further, to achieve an objective comprehension of CH, we studied patients in the headache attack remission state with normal controls.

Our findings in the acute spontaneous CH attack state showed that the altered rs-FC of the hypothalamus with some brain regions is involved in the processing and modulation of pain, referred to as the pain matrix [26], and/or is involved in cognitive and emotional modulation of pain [27]–[29], and some other regions. Many neuroimaging studies of CH have shown that the brain activation is presented in brain regions that are related to pain processing, and/or the affective and cognitive aspects of pain processing, including the ACC, the PCC, the insula, the parahippocampal gyrus, the hippocampus, the amygdale, and the frontal and parietal cortex [4], [8], [12], [30]–[33]. All of these brain changes were observed in other general pain neuroimaging studies [26], [27]. The ACC is a key structure of pain processing. It is not only involved in the evaluation and integration of sensory, cognitive and emotional aspects of pain, as well as in antinociception [27], [34], [35], but also in affective and attentional modulation of pain perception [36]. PCC activation has been linked not only to the processing of pain [37], but also to motor response or its inhibition [38]. It has been suggested that the insular cortex is correlated with subjective pain experience and noxious stimuli [39], [40], and is involved in autonomic reactions to noxious stimuli and in learning and memory related to pain [41]. The frontal, parietal and the temporal lobes are also involved in pain processing and the emotional modulation of pain as some parts of pain matrix [26]. It is inferred that these brain regions may be involved in the pain pathogenesis of CH. Moreover, the hypothalamus has been shown to be the generator of CH [4], [5]. The results of the increased correlation between the right hypothalamus and such brain areas during the spontaneous CH attacks in this study suggest the presence of distributed dysfunction of brain information processing, which may be related to the pain processing and emotional modulation of spontaneous CH attacks.

We also wish to gain some insight into the initial generation of CH attacks by the altered rs-FC of the hypothalamus between CH patients and normal controls. An increased rs-FC of the hypothalamus with parts of the frontal, parietal and temporal cortex was also found in CH patients during headache-free intervals, but not the ACC or the PCC. This suggests that there may be functional abnormalities of pain information processing in CH patients compared to the healthy controls. Furthermore, the increased rs-FC of hypothalamus with the ACC and PCC during the acute spontaneous CH attacks might be the consequence of CH attacks. Alternatively, it may be due to the generation of analgesics because the ACC exerts a descending modulation to gate pain [42] and its activation has been related to analgesia [43]. In addition, a decreased rs-FC of the hypothalamus was found in the occipital cortex beyond the pain-related regions in CH patients. This was also detected in another RS-fMRI study on CH [15]. As it has been reported that many CH patients have photophobic symptoms [44]–[46], photophobia might be related to the altered FC that is associated with the occipital cortex. Unfortunately, we did not collect the photophobia information. Further studies are needed to evaluate this suggestion.

Some caution should be exercised with our results because of the relatively small number of subjects who were enrolled in our study and the limitations of the RS-fMRI method [47]. The individually altered rs-FC for each FC-ROI with the high sensitivity and specificity (all greater than or equal to 83.33%) supports the presence of a brain network dysfunction in CH patients, especially, the individually altered rs-FC between the CH patients and healthy controls. They reflect the goodness of fit of a subject’s default-mode network to a standard default-mode template, which might provide a method to distinguish individual CH subjects from healthy subjects. In addition, it should be noted that the gender selection bias might influence the generalization of the conclusion because only male patients were enrolled in this study. Considering that CH is a predominantly male headache disorder with a 9∶1 ratio of males to females [2], the results might at least reflect to some extent the rs-FC changes of CH during different states.

In conclusion, our findings show that CH patients have a diffuse dysfunction of brain functional connectivity of the hypothalamus. It is mainly in the brain regions that are related to pain processing and modulation during the spontaneous CH attacks, and mainly in the brain regions that include the pain system and visual system during CH attack intervals. Moreover, these changes might be used separately to distinguish the different state of CH patients, as well as CH patients from normal controls.
